# Prevalence and determinants of hand hygiene behavior among Indian population: a systematic review and meta-analysis

**DOI:** 10.1038/s41598-024-52444-2

**Published:** 2024-01-31

**Authors:** P. V. Hareesh, Eslavath Rajkumar, Aswathy Gopi, N. V. Sri Lakshmi K, John Romate

**Affiliations:** 1 Department of Psychology, Central University of Karnataka, Gulbarga, Karnataka India; 2 Department of Liberal Arts, Indian Institute of Technology Bhilai, Durg, Chattisgarh India

**Keywords:** Psychology, Health care, Risk factors

## Abstract

Despite a global call to action, many deaths occur yearly in developing nations from contagious diseases due to poor sanitation and hygiene. Although hand hygiene (HH) behavior was critical in preventing the COVID-19 pandemic, the sustainability of such practices is still questionable. Therefore, the current systematic review and meta-analysis investigated the prevalence and determinants of HH behavior among the Indian population (PROSPERO registration ID: CRD42022344961). Systematic searches on electronic databases, including ScienceDirect, Scopus, Web of Science, JSTOR, PubMed, and Google Scholar, targeted qualitative and quantitative studies that report HH behaviors in India. Pooled effect sizes were calculated with the inverse-variance method using random-effects models, acknowledging the study heterogeneity. Out of 1053 studies, 15 studies that met eligibility criteria were included in the qualitative synthesis. Among them, five studies were included in the meta-analyses. The overall prevalence of HH before food was 55% (95% CI = 31–78), and after the toilet was 84% (95% CI = 65–96). Subgroup analysis showed that before-food HH prevalence pre- and post-COVID-19 was 61% and 36%, respectively, whereas after-toilet HH prevalence was 91% and 74%, respectively. Meta-regression revealed statistically non-significant results for COVID-19 status. While it could not adequately explain the heterogeneity of the ‘before-food prevalence’ studies (Adj. R^2^ = − 34.80%), it did account for more than 19% in ‘after-toilet prevalence’ (Adj. R^2^ = 19.72%). This systematic review highlights various demographic, psychosocial, and environmental determinants of HH behavior. The results offer the potential for a deeper comprehension of the key factors influencing HH in India and could find implications for developing viable interventions. This aids in planning efficient promotional campaigns to enhance personal hygiene and control infectious diseases in the nation.

## Introduction

The prevention and control of contagious diseases continue to be a worldwide concern^1,2^ and there has been a global demand for action to address diseases due to lack of water, hygiene, and sanitation^3^. Maintaining clean hands through better hand hygiene (HH) is one of the essential measures for avoiding illness and transferring germs to others^4^. HH involves washing hands with water and soap or disinfecting them to remove bacteria, viruses, and other microorganisms, as well as grease, dirt, and various undesired and hazardous matters that have adhered to the hands^5,6^. Similarly, good hand washing refers to the act of hand washing using soap and water before eating food and after defecation^7^. The literature has reported a link between better HH and a decline in the rates of infectious diseases^8^. Specifically, hand washing with soap at critical times has reduced acute respiratory infection and diarrhea disease^1,9,10^.

Researchers have revealed a substantial increase in the hand washing behavior of individuals after the COVID-19 outbreak. The WHO identified frequent hand washing with soap and water or cleansing with an alcohol-based hand rub as the first crucial step in protecting oneself and others from COVID-19 infection. HH, on the other hand, is essential not only during a pandemic but also in daily routines. Notably, it is an economical approach to lowering the pressure on a country’s healthcare system and lessening the global burden of diseases^11^. However, despite proven effectiveness, HH compliance remains low worldwide^12^, particularly in developing countries.

A national survey conducted in India in 2018 identified that only 35.82% of the population regularly follows HH before meals^13^. Poor post defecation hand washing and anal hygiene practices are widespread in the Indian subcontinent and are key contributors to the fecal–oral transmission of enteric illnesses^14,15^. As a result, enhancing HH behaviors is a crucial need of the country to prevent and control infectious diseases. Moreover, an integrated strategy incorporating better personal hygiene and concurrent expansion of public health infrastructure can successfully manage infections^8,16,17^. Thus, it is crucial to delve into the prevalence of HH behaviors and the factors that determine them in India, a country with a population density of 464 sq. km^18^. In addition, according to the provisional population estimates of the 2011 census, the country’s population density has increased from 325 persons per sq. km in 2001 to 382 persons per sq. km^19^, which highlights the need for promoting personal hygiene to prevent infections among the Indian population.

Although several studies report the prevalence and factors influencing HH behavior in India, no studies compile such evidence to comprehensively present the rate of HH practices in the country. Thus, the present study aimed to provide a pooled estimate of HH behavior and the determinant factors among the Indian population. Hence, this study made an effort to address the following research questions. (1) What is the prevalence of HH behavior among the Indian population? (2) What factors determine HH behavior among the Indian population? The review findings may guide governmental and non-governmental agencies, healthcare authorities, researchers, and other stakeholders to develop and implement effective infection prevention strategies in the country.

The aim of the current study is to find the prevalence and determinants of HH behavior among the Indian population.

## Methods

The systematic review and meta-analysis were reported following the Preferred Reporting Items for Systematic reviews and Meta-Analyses (PRISMA) (see Supplementary Table S1) and registered in PROSPERO with the ID: CRD42022344961.

### Eligibility criteria

In the present study, HH behavior refers to hand washing with water and soap or alcohol-based hand rub and other hand disinfection practices to kill germs and bacteria. As per the PICOS guidelines, the study eligibility criteria included the following: (a) Population: studies focusing on the Indian population of any age and gender were included. (b) Intervention/exposure: studies reporting individual HH behaviors were eligible for selection. (c) Comparison: not applicable in the current study. (d) Outcomes: studies targeting the prevalence of HH behaviors and the determinants of such behaviors were included. (e) Study design: Qualitative and quantitative studies were considered for the systematic review. Cross-sectional studies that reported the prevalence of HH behavior were included in the meta-analysis. Both peer-reviewed quantitative and qualitative investigations were included, while editorials, commentaries, and reviews were excluded. There was no limit kept on the year of publication. However, the search was restricted to studies published in the English language.

### Information sources and search strategy

The PubMed, JSTOR, ScienceDirect, Web of Science, Scopus, and Google Scholar electronic databases were systematically searched for studies published on the prevalence and determinants of HH behavior among the Indian population. The reference lists of related studies were also examined. The search was performed in September 2022. The searches were rerun before the final analysis to identify further relevant studies. To identify the articles, the systematic search employed the following keywords: “hand hygiene,” “hand wash,” “hand disinfection,” “hand sanitizer,” “hand rub,” “prevalence,” “percentage,” “determinants,” “factors,” and “India.” Boolean operators (AND/OR) were used to combine the terms in each database. The entire search strategy used for each electronic database can be found in Supplementary Table S2.

### Selection process

All identified records were exported into Zotero reference management software^20^ for duplicate verification. After deduplication, the titles and abstracts of the remaining records were manually screened by three reviewers (P.V.H., E.R., and A.G.) in Microsoft Excel and irrelevant publications were excluded. Consequently, two reviewers (P.V.H. and A.G.) independently screened the full text of studies that met the eligibility criteria. Disagreements during the screening process were settled through discussion or involving a third reviewer (E.R.).

### Data collection process

Following the study inclusion criteria, two reviewers (P.V.H. and A.G.) independently exported significant information from all eligible studies to Microsoft Excel. The extracted data included the author(s) name, publication year, study design, region, sample characteristics, prevalence, and determinants. The author(s) name, year of study, prevalence of HH before food, prevalence of HH after toilet, and sample size were retrieved for meta-analysis. The prevalence of HH before food included all the prevalence before the intake of meals, dinner, or any other food, and the prevalence of HH after the toilet included any HH behaviors after using the toilet, after defecation, after urination, etc. If multiple prevalence were given in the same study for the outcome mentioned in the above domains, their average value was taken for the analysis. Disagreement between the investigators about study eligibility was resolved through discussion or consultation with a third reviewer (E.R.).

### Study risk of bias assessment

Three reviewers (P.V.H., A.G., and N.V.S.K.) independently appraised the quality of each selected study using the JBI critical appraisal checklists (see Supplementary Figs. S1, S2, S3). Depending upon the study design, tools for analytical cross-sectional studies, cohort studies, and qualitative research were employed. Discrepancies in the quality assessment process were resolved by discussion among authors or consultation with a fourth reviewer (J.R.).

### Data synthesis and meta-analysis

A narrative synthesis of extracted information on determinants of HH behavior and meta-analyses for the prevalence was carried out. The quantitative data was analyzed using STATA 17.0 software^21^ and subsequently presented with forest plots, funnel plots, and bubble plots. The random-effects model was used for meta-analysis due to observed between-study heterogeneity in terms of sample and geographic characteristics. Also, HH behaviors can vary depending on many factors identified in the determinants. The meta-analysis found the pooled effect size and 95% confidence interval through the inverse variance method. The heterogeneity was investigated based on I^2^ values; higher I^2^ indicated higher heterogeneity between the studies. Subgroup analysis was conducted on the factor ‘COVID status,’ which included the studies conducted before and after the outbreak of the COVID-19 pandemic. Then, meta-regression was performed to understand the influence of the variable on the heterogeneity of the overall prevalence. All the results identified were statistically significant if *p* < 0.05.

### Reporting bias assessment

Egger’s test for small study effect and funnel plots were used to assess the publication bias of the included studies in the meta-analysis, where *p* < 0.05 indicates significant publication bias.

## Results

### Study selection

**Figure 1:**
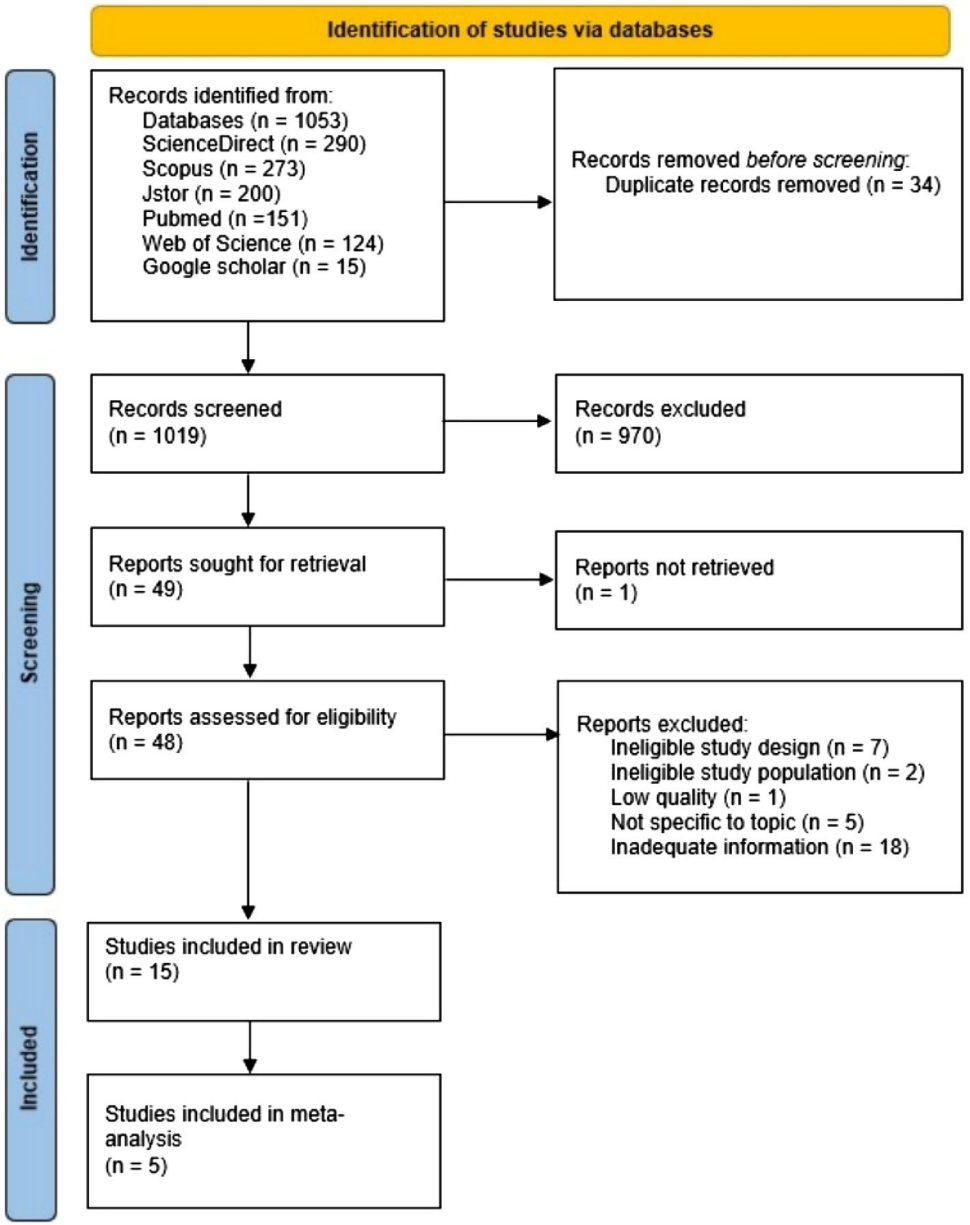
PRISMA 2020 flow diagram for the study selection.

The searches yielded a total of 1,053 studies from six different databases, including 290 studies from ScienceDirect, 273 from Scopus, 124 from Web of Science, 200 from JSTOR, 151 from PubMed, and 15 from Google Scholar. After removing 34 duplicates, initial title/abstract screening was performed for 1,019 studies, excluding 970 records. Subsequently, 49 records were sought for retrieval. Of these, full-text analysis was performed for 48 reports since one study was not accessible. Due to various reasons such as ineligible study design (n = 7), ineligible study population (n = 2), low quality (n = 1), not specific to topic (n = 5), and inadequate information (n = 18), 33 reports were excluded during full-text screening. Finally, 15 studies were included in the systematic review and five in the meta-analysis. The information collected from finalized studies consisted of the author/s name(s), publication year, region, study design, sample age, sample size, sample, prevalence, and determinants. Figure 1 depicts the PRISMA 2020 flow diagram^22^.

### Study characteristics

The current systematic review included 15 studies, and a summary of study characteristics is depicted in Table 1. It also consists of the characteristics of the five studies included in the meta-analysis. Most of the included studies were cross-sectional, with only one prospective cohort study^23^. Six studies targeted children under 18 years as their population, and four focused on healthcare workers working in hospital settings. The age group in the studies ranged from 6 to 84 years old. The total number of samples in the studies selected for the systematic review was above seven lakhs (7,00,596). The lowest number of participants, 64, was reported by Sharma et al.^24^, and the largest sample size of 5,82,064 was included in a study by Pradhan and Mondal^25^. Only one study^23^ was exclusively conducted among females. Few studies (n = 3) were conducted among different countries while also including the Indian population^26–28^. A total of 1,15,285 samples were studied in the meta-analysis. Of these, the study by Gupta and Anand^29^ had the smallest number of samples (N = 106) and the largest number in the study by Biswas and Karmakar^30^ (N = 1,06,837). Among the studies sought for meta-analysis, three studies were conducted before the COVID-19 pandemic^27,31,32^ and two studies after the outbreak^29,30^.

**Table 1:** Study characteristics (n = 15).

Study	Region	Study design	Sample age	Sample size	Sample	Determinants	Prevalence of hand hygiene before food	Prevalence of hand hygiene after toilet	Risk of bias
Schmidtke and Drinkwater^28^	India	Cross-sectional	18–70 years	825	Parents of children 5 to 10 years old and additional 75 teachers	Capability, which includes knowledge, skills, memory, attention and decision processes, and behavioral regulation; opportunity, which includes environmental contexts and resources’ social influences	–	–	Moderate
Ray et al.^32^	Bangalore and Kolkata	Cross-sectional	9–19 years	208	Students in 6th to 8th classes	Perception of dirty areas of hands, knowledge regarding the importance of hand washing, availability of soap at hand washing facility	86% (179/208)	97.6% (203/208)	Moderate
Biswas and Karmakar^30^	India	Cross-sectional	–	1,06,837 (Households)	Residents of India	Place of residence, religion, social group, level of education of household head, wealth status, family size, benefits received from the government on schemes for drinking water or related to sanitation, access to the principal source of drinking water and water for all household activities, access of household to the bathroom, availability of bathrooms and latrines, availability of water in or around the latrine used	35.82% (38,269/106,837)	74.15% (79,220/106,837)	Moderate
Garg et al.^23^	Delhi	Cohort study	10–16 years	281	Girl students from 6 to 8th class	Knowledge concerning the importance of hand washing, knowledge about diseases transmitted through contaminated hands, and technique of hand washing	–	–	Moderate
Seimetz et al.^34^	Rural parts of Northern India	Cross-sectional	16–84 years	687	Participants with an age of at least 16 years	Risk factors, which is perceived vulnerability, attitude factors, which include instrumental belief response, the affective belief of liking and dirtiness, norm factors, which is the injunctive norm; ability factors which are action self-efficacy; and self-regulation factor, which is the commitment	–	–	Moderate
Pati et al.^31^	Odisha	Cross-sectional	6–55 years	230 (150 Women, 80 children)	Urban slum children and their caretakers	Availability of soap, perceived susceptibility to diarrhea, knowledge regarding the relationship between hand washing and prevention of infections, awareness among children about hand hygiene	35.2% (81/230)	68.26% (157/230)	Moderate
Sharma et al.^35^	Punjab	Cross-sectional	21–60 years	114	Health care workers, including all physicians on rounds in intensive care units (ICU), intensivists, all postgraduate residents, and nurses in ICUs	Workload, opportunity (potential hand hygiene action needed), lack of knowledge, time, lack of motivation, lazy attitude, administrative apathy	–	–	Moderate
Sharma et al.^24^	Chandigarh	Cross-sectional	–	64	Healthcare workers in Intensive Care Unit (ICU)	Lack of time, workload pressure, lack of knowledge about the importance of hand hygiene, lack of institutional commitment, and availability of soap in the washing area	–	–	Moderate
Ranasinghe et al.^27^	India	Cross-sectional	13–15 years	7,904	Students in 8th, 9th and 10th grades	Depression and loneliness	–	96.66% (7640/7904)	Moderate
Pradhan and Mondal^25^	India	Cross-sectional	–	5,82,064	Households in India	Education of household head, female-headed households, size of households, social status, lack of knowledge, availability of water, wealth status	–	–	Moderate
Anderson-Carpenter and Tacy^26^	India	Cross-sectional	18 years and above	500	Residents of India	Urbanicity, age, gender	–	–	Moderate
Joshi et al.^37^	Ujjain	Cross-sectional	18–68 years	75	Hospital staff of rural teaching hospital in India	Perceived importance of hand hygiene, the feasibility of hand washing, type and material of hand hygiene products, scarcity of water, number of washbasins, the distance of wash basins from patients	–	–	Moderate
Dobe et al.^7^	West Bengal	Cross-sectional	10–19 years	442	Adolescents in the rural Indian community	Presence of Sanitary Latrine, in-house water supply, availability of soap at washing location, education, dwelling, economic status	–	–	Moderate
Diwan et al.^33^	Madhya Pradesh	Cross-sectional	19–70 years	259	All healthcare workers and nursing students at teaching hospitals in rural India	High workload, lack of time, scarcity of resources, lack of scientific information, and perception of the importance of hand hygiene	–	–	Moderate
Gupta and Anand^29^	Haryana	Cross-sectional	10.2 years of mean age	106	5th standard students in rural government primary schools	Poor illness threat perception, poor attitude towards hand hygiene, doubts on outcome expectation, motivation through a feeling of disgust, visibility of dirt, peer influence, the distance of hand washing station	59% (63/106)	67% (71/106)	Moderate

### Risk of bias in studies

The risk of bias in the studies was assessed using the appropriate JBI critical appraisal checklists. Sixteen studies that met the eligibility criteria were evaluated for quality including 13 cross-sectional studies^7,24–35^, two cohort studies^23,36^ and one qualitative study^37^. All the cross-sectional studies had a moderate risk of bias/quality after the assessment. Among the cohort studies, Garg et al.^23^ had a moderate risk of bias, while Taneja and Mishra^36^ had a high risk of bias. This indicates that the latter has low quality, and therefore, it was excluded from further analysis. The qualitative study^37^ had a moderate bias risk. There were no high-quality studies in the reports. The quality assessment results can be found in Supplementary Tables S3, S4, S5.

### Prevalence of HH behavior

**Figure 2:**
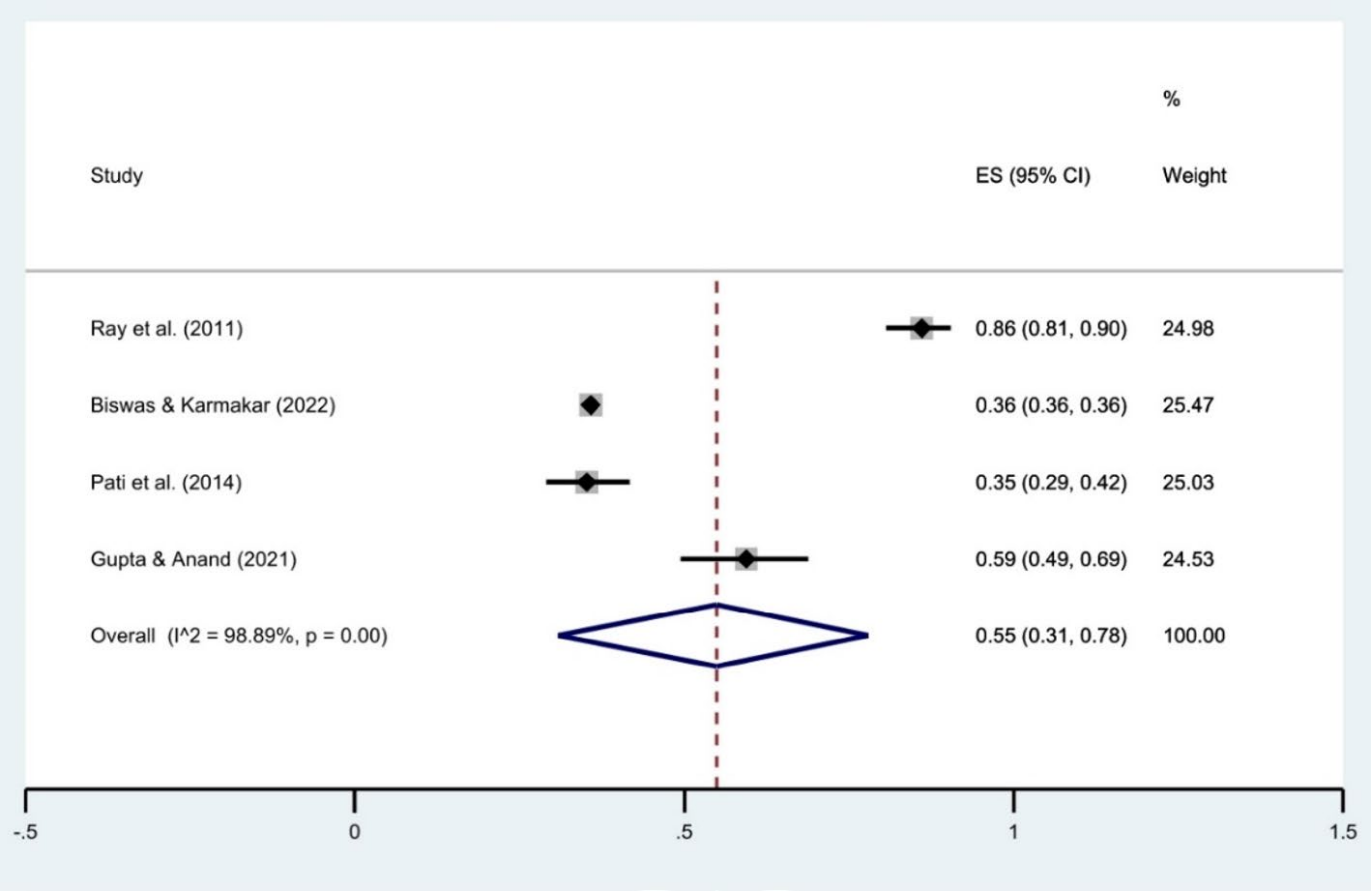
Forest plot showing the overall prevalence of HH behavior (before food). CI = Confidence Interval; I^2^ = I^2^ statistic for measuring heterogeneity.

**Figure 3:**
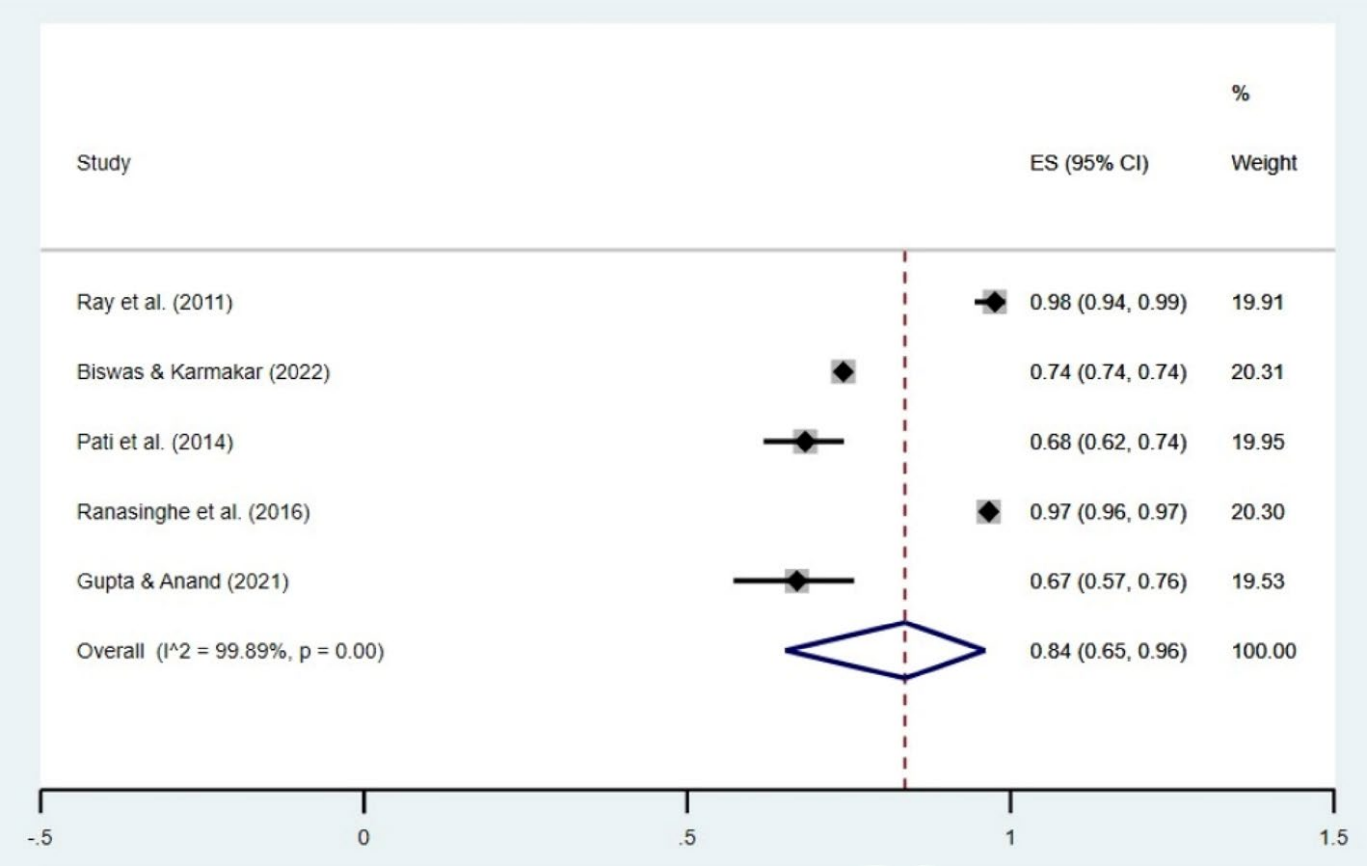
Forest plot showing the overall prevalence of HH behavior (after toilet). CI = Confidence Interval; I^2^ = I^2^ statistic for measuring heterogeneity.

The random effect meta-analysis revealed a pooled prevalence estimate of 55% (95% CI 31–78) for HH behavior before food (Fig. 2) and 84% (95% CI 65–96) after toilet use (Fig. 3) within the Indian population. Studies on HH before food showed high heterogeneity (I^2^ = 98.89%), and those on HH after the toilet exhibited substantial heterogeneity (I^2^ = 99.89%). All findings were significant (*p* < 0.001).

### Subgroup analysis

Table 2 indicates the prevalence of HH behavior before food and after toilet in studies conducted before the COVID-19 outbreak.

**Table 2:** Prevalence of HH behavior and results of subgroup analysis.

Context	Subgroup	No. of studies	N	p (%)	95% CI	I^2^ (%)	*p*_1_
Hand hygiene before food	Before outbreak	2	438	61*	(56, 66)	–	0.675
	After outbreak	2	1,06,943	36*	(35, 36)	–	
Overall		4	1,07,381	55*	(31, 78)	98.89*	–
Hand hygiene after toilet	Before outbreak	3	8342	91*	(72, 100)	98.71*	0.274
	After outbreak	2	1,06,943	74*	(74, 75)	–	
Overall		5	1,15,285	84*	(65, 96)	99.89*	–

*p < 0.001.

^a^n = total sample size, p = prevalence, CI = confidence interval, I^2^ = residual heterogeneity static, *p*_1_ = *p* value for meta-regression.

### Prevalence of HH before food, based on COVID-19 status.

**Figure 4:**
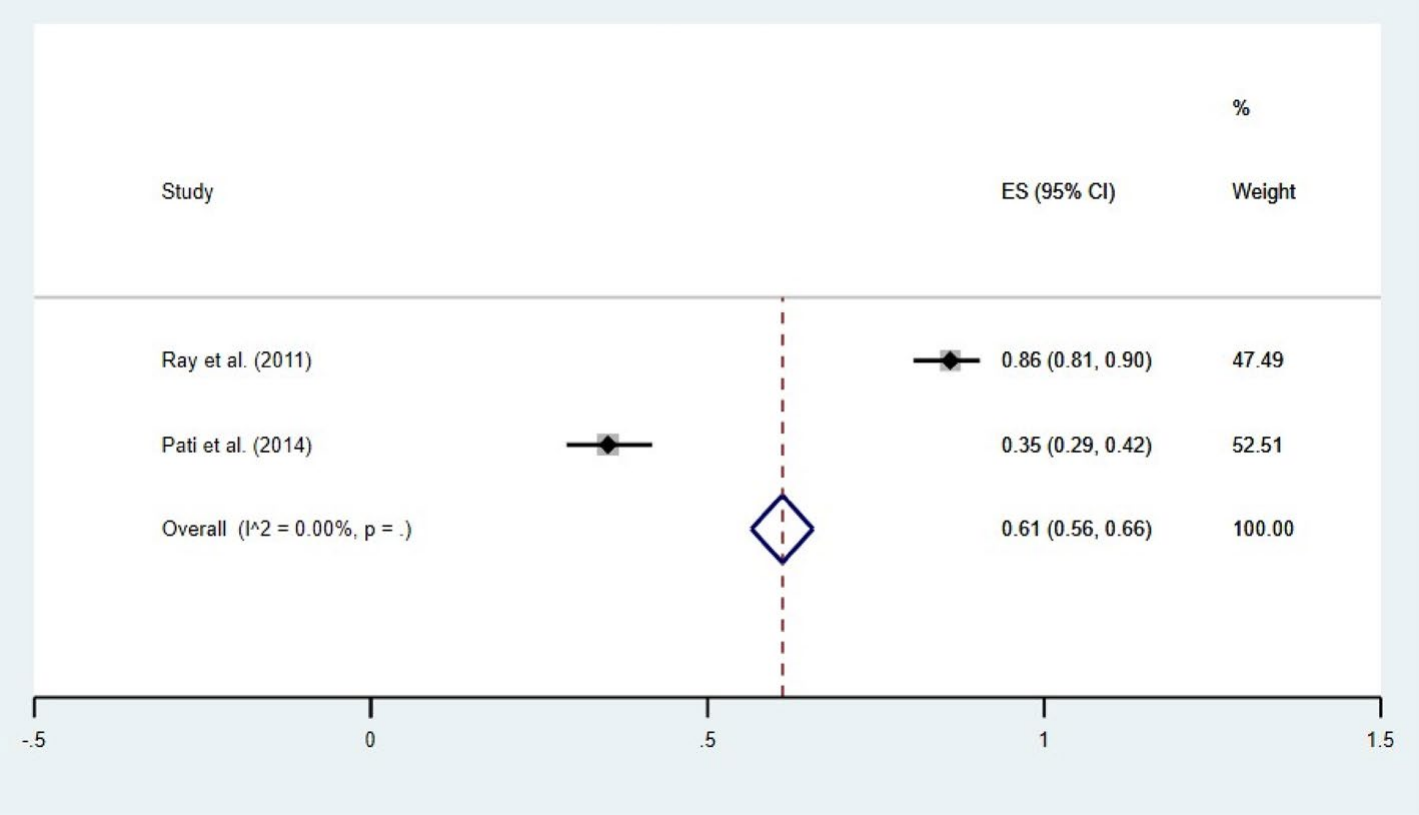
Forest plot showing prevalence of HH behavior before COVID-19 outbreak (before food). CI = Confidence Interval; I^2^ = I^2^ statistic for measuring heterogeneity.

**Figure 5:**
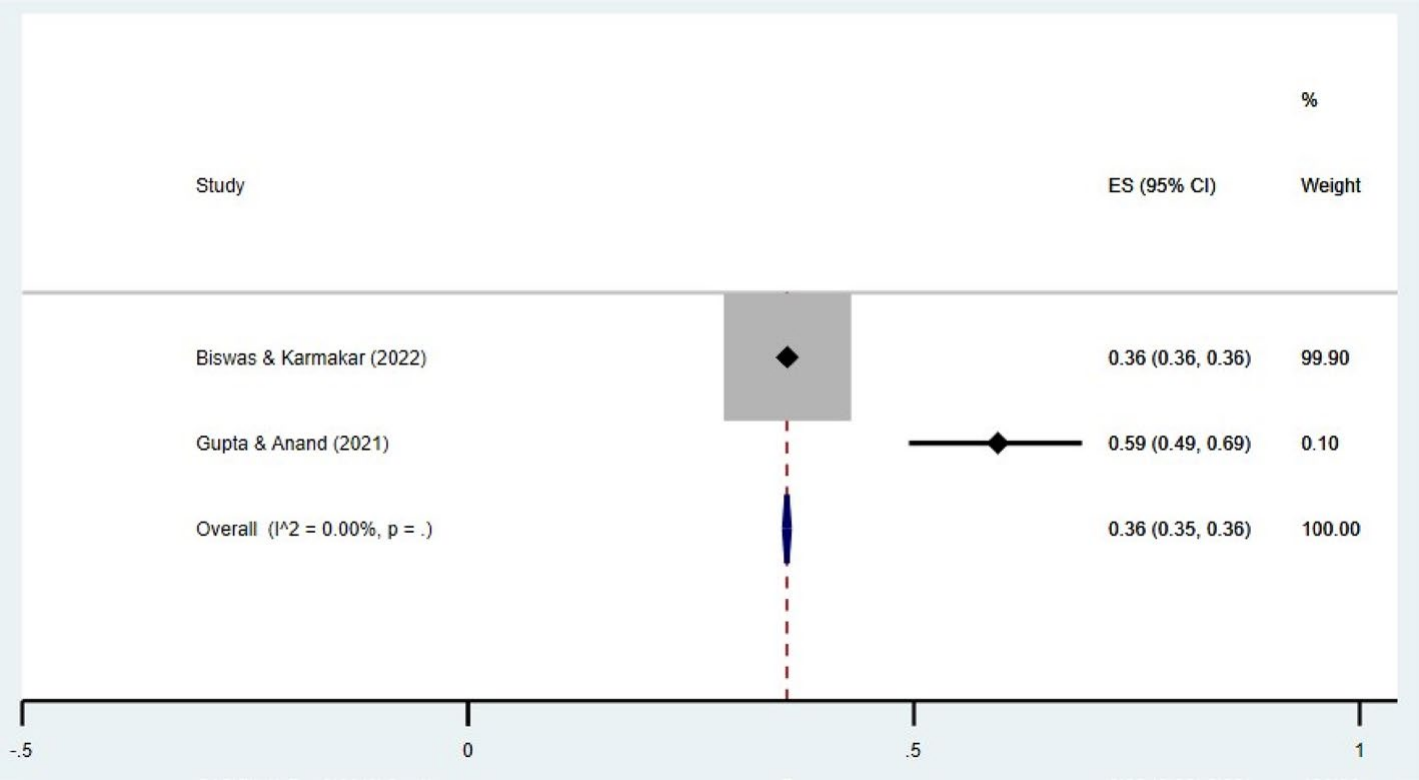
Forest plot showing prevalence of HH behavior after COVID-19 outbreak (before food). CI = Confidence Interval; I^2^ = I^2^ statistic for measuring heterogeneity.

The subgroup analysis revealed that before the COVID-19 pandemic, the prevalence of HH behavior before food was 61% (95% CI 56–66), and after the pandemic, it was 36% (95% CI 35–36). The forest plots depicting these results are given in Figs. 4 and 5.

### Prevalence of HH after toilet, based on COVID-19 status

**Figure 6:**
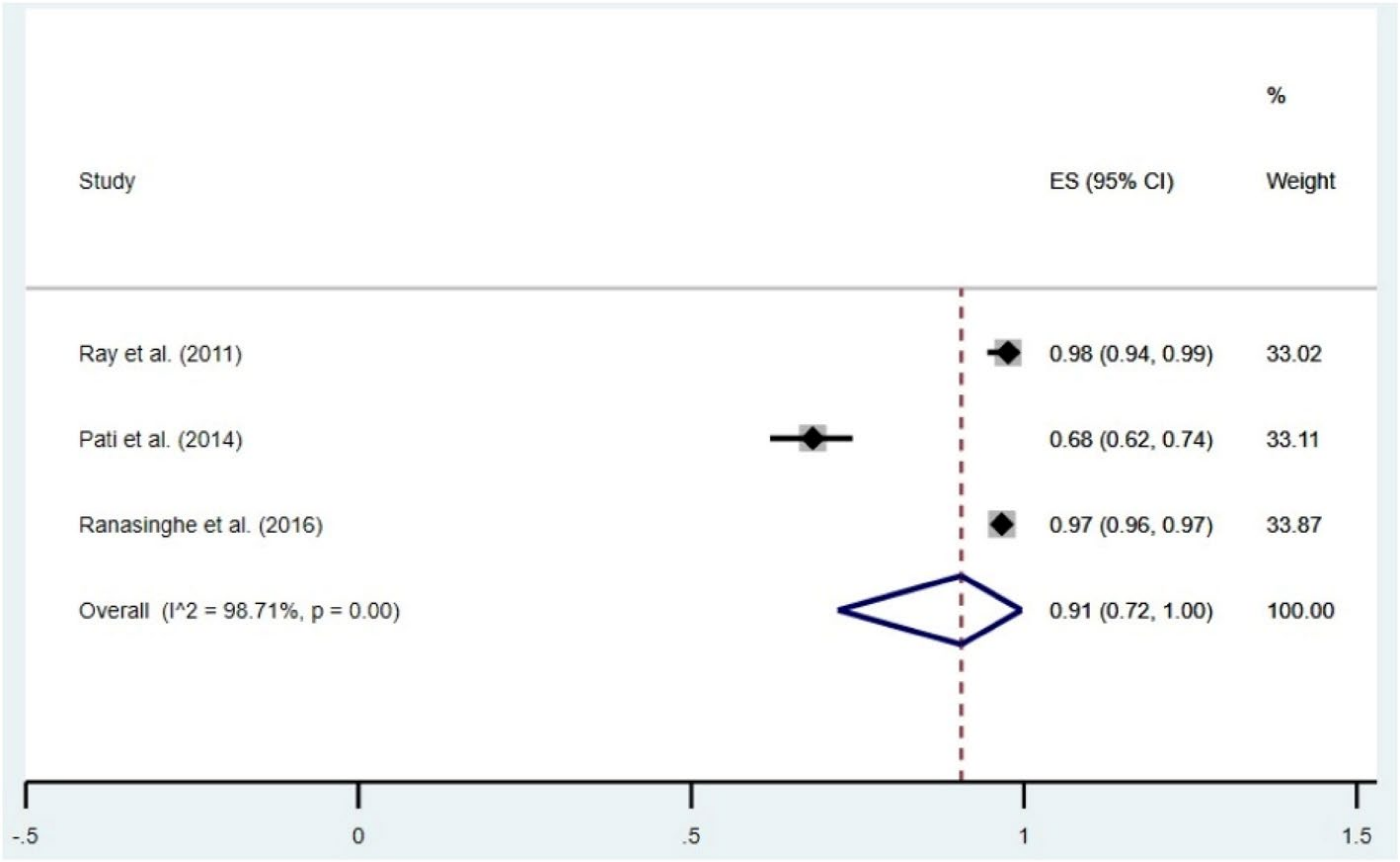
Forest plot showing prevalence of HH behavior before COVID-19 outbreak (after toilet). CI = Confidence Interval; I^2^ = I^2^ statistic for measuring heterogeneity.

**Figure 7:**
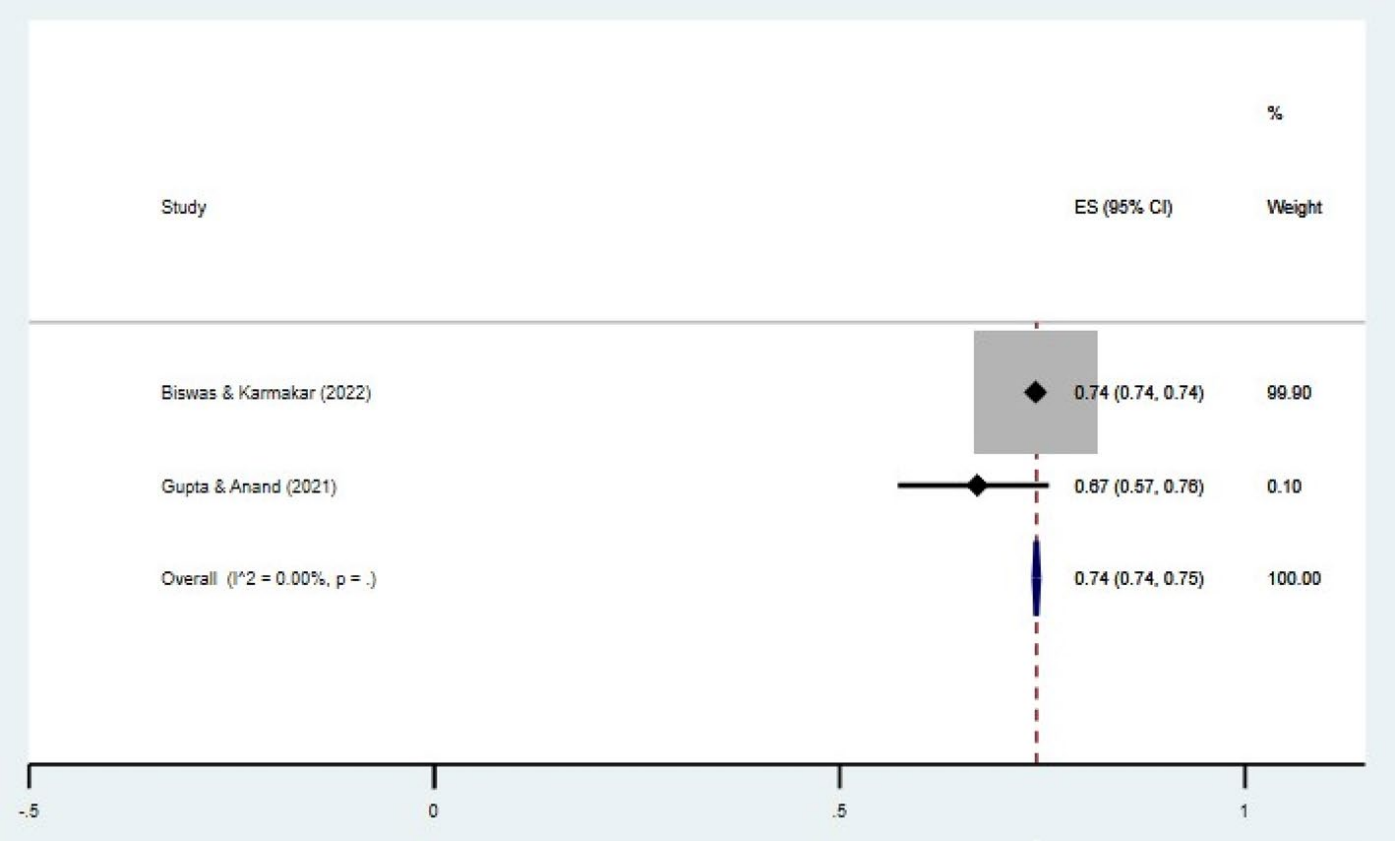
Forest plot showing prevalence of HH behavior after COVID-19 outbreak (after toilet). CI = Confidence Interval; I^2^ = I^2^ statistic for measuring heterogeneity.

The prevalence of HH behavior after the toilet, before the COVID-19 outbreak, was 91% (95% CI 72–100). The prevalence of the same after the COVID-19 outbreak was 74% (95% CI 74–75). The forest plots depicting these results are given in Figs. 6 and 7.

### Meta-regression

The random effect meta-regression results (Table 3) revealed that the heterogeneity/variance between the studies (adjusted R^2^) with before-food prevalence was not well explained by COVID-19 status (see Supplementary Figs. S4, S5). In contrast, more than 19% of heterogeneity among studies with after-toilet prevalence was explained by COVID-19 status. The residual heterogeneity static (I^2^_res_) shows that 94% of the residual variance before food prevalence studies was due to heterogeneity, and the remaining 6% was due to the within-study sampling variability. In the after-toilet prevalence studies, almost 84% of the residual variance is due to heterogeneity. However, the meta-regression results for both prevalence studies were not statistically significant (*p* > 0.05), which could be attributed to the fewer studies used in the analysis for each context.

**Table 3:** Meta-regression on COVID-19 status.

Characteristic	Context	No. of observations	I^2^_res_ (%)	Adj. R^2^ (%)	*p* > |t|
COVID-19 status	Before food	4	94.16	− 34.80	0.675
	After toilet	5	83.93	19.72	0.274

^a^I^2^_res_ = residual heterogeneity static, Adj. R^2^ = corrected goodness-of-fit measure, *p* = *p* value.

### Demographic determinants of HH

#### Age

One study identified age as a determinant of HH behaviors and reported that older people are more likely to follow proper HH practices than younger people due to their perception of a higher risk of infection vulnerability^26^.

#### Gender

Two studies showed that females were more involved in HH behaviors than males^25,26^. Especially when they are family caregivers, females prioritize HH practices to avoid infections. Pradhan and Mondal^25^ attributed the HH compliance of females to their better knowledge about the importance of hand washing.

#### Education

Three studies^7,25,30^ identified that education level is a determinant of HH practice. The households with high education levels for the head of the house reported a better rate of HH practices than those with lower education levels.

#### Place of residence

Three studies^7,26,30^ reported that people living in rural areas and more crowded areas have a lower rate of HH behavior. These studies highlight an association of area of living with knowledge and access to hygiene resources, as people living in urban areas had better access to these resources.

#### Wealth status

Three studies associated higher wealth status with a better rate of HH practices^7,25,30^. Specifically, the lower economic population reported less affordability to obtain hygiene products for daily use.

#### Family size

Two studies^25,30^ showed family size as a determinant for following HH behavior. The greater the number of members in the family, the greater the rate of HH.

#### Religion

Only one study^30^ found that religion was associated with HH. It was found that people of Hindu and Christian religions followed hand washing with soap before having food more likely than Muslim religion people. On the other hand, people from the Muslim community had better hand-washing behaviors with soap than the other two religions after defecation.

### Psychosocial determinants of HH

#### Motivational factors

The role of motivation in compliance with HH behavior was identified by two studies^29,35^. In their study, Sharma et al.^35^ found that healthcare workers lacked the motivation to perform HH. Conversely, a study among upper primary students by Gupta and Anand^29^ found that students showed more motivation to hand wash with soap, which was induced by disgust after defecation. Relatedly, two studies^29,37^ identified that the longer distance to reach a hand-washing area was a barrier to motivating people to wash their hands at regular intervals. Similarly, poor outcome expectancy was associated with HH behavior^29^.

#### Attitudinal factors

A study by Seimetz et al.^34^ based on the Risk, Attitudes, Norms, Abilities, and Self-regulation (RANAS) model of systematic behavior change identified the domains in attitude, such as instrumental belief, the affective belief of liking, and dirtiness, as significant determinants of HH practice among the Indian population. Sharma et al.^35^ reported that healthcare workers reported lazy attitudes in HH behaviors attributed to workload pressure and lack of time during work. In a study, students revealed that their poor attitude toward considering it okay to skip hand washing before eating significantly reduced their compliance with HH^29^.

#### Normative factors

Social norms concerning acceptable behavior can influence most individual behaviors. One study^34^ found that injunctive norms determine HH among the Indian population.

#### Ability factors

Two studies revealed that people are more likely to adopt HH behavior if they are confident in their ability to do so^28,34^. Seimetz et al.^34^ identified self-regulation as a determinant of hygiene practices where individuals who identified themselves with HH behavior and showed better commitment followed more HH practices.

#### Peer influence

The study by Gupta and Anand^29^ demonstrated that children receive knowledge and awareness regarding the benefits and techniques of HH from their peer groups. Children are more likely to follow knowledge that has been shared with them by teachers, parents, and other peer groups.

#### Workplace factors

Three studies^24,33,35^ conducted among healthcare workers revealed that workload pressure made it difficult to perform HH behaviors. In addition, two studies^24,35^ reported that a lack of commitment and apathy toward HH from healthcare institution administration reduced healthcare workers’ compliance.

### Environmental determinants of HH

#### Opportunity

A study by Schmidtke and Drinkwater^28^ found that the opportunity factor in the COM-B model (capability, opportunity, and motivation) of behavior was significant in determining the HH behavior of the Indian population. This factor consists of domains such as environmental contexts, resources, and social influences. Moreover, most studies (n = 10) reported the role of opportunity as a determinant.

#### Visible cues

Two studies^29,32^ that targeted children revealed that either they did not have the perception of dirt getting the nails or web spaces of hands or they wanted proof of visible dirt/germs in the hand. No study among the adult age group found this factor.

#### Type and material of the product

The study conducted by Joshi et al.^37^ found that the type and material of hygiene products used for hand washing can be a determinant of HH behavior. Some people might be allergic to certain chemicals, while others refuse to use soap and other sanitizing products because of their smell.

### Publication bias

The funnel plots for before-food HH prevalence studies (see Supplementary Fig. S6) and after-toilet HH prevalence studies (see Supplementary Fig. S7) showed an asymmetry in the distribution that suggests a chance of publication bias. This was clarified in Egger’s test for the small study effect. The test revealed a nonsignificance (*p* > 0.05), confirming there were no missing data in the publication of the studies (see Supplementary Table S6).

## Discussion

Much research has undoubtedly pointed out the importance of following HH behaviors in different instances^38,39^. HH is an important topic to be discussed, focused on, and followed in daily life to efficiently reduce the spread of many infectious microorganisms. To the best of the authors’ knowledge, no other reviews have been conducted on HH among the Indian population. Contradictory to existing findings^14,15^, the current study found a high prevalence of HH behavior in the country. On the other hand, a statistically significant decrease in prevalence after the pandemic remains a concern for future research. However, given the limited number of studies and heterogeneity, evidence must be considered with caution. Furthermore, the present review identified various demographic, psychosocial, and environmental determinants of HH behavior.

The prevalence of HH behavior was high after using the toilet, which is substantially greater than the global prevalence (19%) reported in an earlier study^40^. This might be because people perceive dirtiness after using the toilet or defecation, which prompts them to engage in HH behavior^29,32^. The lack of resource availability was a factor that reduced HH behaviors among health workers in China^41^. This also became a part of the injunctive norms in Indian society^34^. In this regard, there is also a higher chance of resources such as the availability of soap or enough water supply in the toilets, which adds to the following of HH^7,24,25,31,33,37^.

Past literature revealed that the COVID-19 pandemic made HH a frequent practice, and people largely started HH behaviors^42^. Relatedly, a study conducted in Indonesia found a significant increase in the practice of HH by people after the outbreak^43^. However, the subgroup analysis on ‘COVID-19 status’ in the present study provides inconsistency with these findings. Interestingly, in both contexts, such as before food and after toilet, the prevalence of HH decreased significantly after the COVID-19 outbreak. In 'before food prevalence' investigations, HH prevalence was decreased by nearly half. This drop in the prevalence might be attributed to fewer studies and a large variation in sample size. Nevertheless, one cannot reject these findings since they are a major public health concern.

The determinants of HH behavior included demographic, psychosocial, and environmental factors. Demographic factors such as age, gender, education, place of residence, wealth status, family size, and religion were associated with HH behaviors among the Indian population. The findings are consistent with a similar systematic review conducted in Turkey^44^ that reported the influence of gender on HH behavior.

The present study identified psychosocial factors such as motivational, attitudinal, normative, ability, peer influence, and workplace factors as determinants of HH. For instance, two studies^29,31^ revealed that the poor perception of the consequence of illness as a threat, especially by the younger population (< 18), reduces perceived susceptibility, and they had low compliance with HH. This corroborates the findings of a similar systematic review^45^ on hygiene behavior, which was conducted based on the Risk, Attitudes, Norms, Abilities, and Self-regulation (RANAS) structure.

A related study among nurses in United States hospitals revealed certain factors that comply with the results of the current review, such as injunctive norms, workload pressure, administration at the workplace, and time^46^. Similarly, a study among care providers in community clinics in Bangladesh^47^ identified the determinants of HH, such as availability and type of hygiene products, knowledge/HH training, and visible cues. However, these results are more relevant to people working in hospital settings or places that demand better hygiene protocols. The current findings are in line with a study that reported that the determinants of HH behavior among staff in veterinary referral practices in the United Kingdom were time constraints and access to equipment. However, their results contradicted self-reported responses including social norms, self-protection, habits, and patient protection^48^. The authors suggested this could be due to social desirability that promotes the staff to view the factors to be significant in the clinic even when they are not determining their HH behavior.

The findings highlight that when access to hygiene supplies and infrastructure was limited, the chance for HH was reduced. Ethiopian research found that the availability of resources has a significant influence on HH compliance^49^. This emphasizes the need for concerned authorities and government agencies to collaborate in making resources available to the public. These results suggest that most of the present findings corroborate the general findings on HH behavior across the world.

The studies included in the current review have certain methodological concerns. In six out of 15 studies^24,25,27,28,32,34^, the sample inclusion criteria were unclear and a majority (n = 12) did not report any information regarding confounding factors and strategies to control them. One study^33^ did not follow a standard criterion for measuring the HH prevalence while another two^25,30^ did not measure the exposure similarly among the samples. The validity and reliability in the way of measuring HH behavior were not specified in one study^30^. The qualitative study^37^ lacked transparency in locating the researcher culturally or theoretically and there was no statement revealing the influence of the researcher on the research. These methodological restraints need to be addressed in future studies since reliable evidence is inevitable for healthcare policy implications.

The insight into the limitations of the present study underscores the need for cautious interpretation. Most of the studies included in the current analysis were cross-sectional, with only one being longitudinal, limiting the causal inferences regarding the identified HH determinants. Self-report measures were predominantly used in the included studies, which are subject to social desirability and recall biases. Additionally, online recruitment methods employed in some studies may have introduced selection bias, as populations lacking technological infrastructure or digital literacy are excluded.

Differences in the conceptualization of HH were observed among the included studies. There were variations in the tools used to measure HH behaviors and the types of HH practice examined. This could have led to the high between-study heterogeneity which was reflected in the meta-analysis. Furthermore, the diversity in the populations and their requirement for HH behavior are inherently different, thus complicating the comparability of the study findings.

Due to fewer studies reporting quantitative evidence on determinants of HH behavior, a qualitative approach was adopted for data synthesis, which limits the robustness and generalizability of the findings. Moreover, the study population exhibited considerable variation in their sample sizes, sample ages, and geographic areas, leading to a high between-study heterogeneity. The number of studies eligible for meta-analysis was small, thus undermining the power of the meta-analyses and subgroup analysis results. For instance, the prevalence of HH after COVID-19 (before food and after toilet) was dominated by one study, which may have influenced the overall low HH prevalence after COVID-19 when compared to before COVID-19. Furthermore, a paucity of data prevented our review from performing a nationwide regional comparison of HH behavior before and after COVID-19. Nonetheless, the current review adds valuable information to the existing literature on HH behavior.

The current findings offer relevant practical implications for different stakeholders, including the government, healthcare professionals, community leaders, academicians, and employers. Engaging and collaborating with these stakeholders is essential for developing comprehensive and effective HH programs throughout the nation. Government agencies responsible for healthcare regulation and accreditation may set standards and guidelines for HH practices with the support of the current review findings. Being the concerned authority for healthcare, public health departments can develop and implement population-specific interventions to prevent and control infections. Awareness campaigns can highlight the importance of HH before food consumption and after toilet use, emphasizing the role of personal and community health. Additionally, traditional and digital media can be used to disseminate information about strategies for infection prevention. Community leaders and influencers should ensure clean water and sanitation facilities in their respective localities, providing a conducive environment for HH practices. Just as educational institutions are another stakeholder in creating a culture of HH practices during the early developmental phases of children, employers have a responsibility to create a safe and healthy work environment for the well-being of employees. In short, collaborative efforts between international organizations, inter-state organizations and local stakeholders should be encouraged to share best practices and strategies for promoting HH.

Insights from this review add to the understanding of the prevalence and determinants of HH in the Indian population. However, a lack of state-wise exploration of HH practices recommends future research emphasizing the influence of additional factors such as cultural values, demographic characteristics and geographical diversity. Further, future research can focus on rural, socially backward groups and other vulnerable populations because of their limited access to hygiene resources, and inadequate knowledge and awareness of HH practices. A majority of cross-sectional studies in the review sample suggest the need for longitudinal research that helps monitor and understand the underlying causes of HH behaviors.

## Conclusion

The present study highlights the overall prevalence of HH before food and after toilet use among the Indian population. Demographic, psychosocial, and environmental factors were identified as the determinants of HH behavior in the country. Government and other stakeholder initiatives can focus on these factors to improve the success rate of intervention plans focused on the sustainability of HH practices and better management of infections.

### Supplementary Information

Supplementary Information.

## Data Availability

The data that support the findings of this study were derived from the databases (ScienceDirect, Scopus, Web of Science, JSTOR, PubMed, and Google Scholar) available in the public domain.
